# Hepatic recruitment of myeloid-derived suppressor cells upon liver injury promotes both liver regeneration and fibrosis

**DOI:** 10.1186/s12876-024-03245-4

**Published:** 2024-05-14

**Authors:** Qiongwen Zhang, Ting Yu, Huaicheng Tan, Huashan Shi

**Affiliations:** 1 Department of Head and Neck Oncology, Cancer Center, State Key Laboratory of Biotherapy, West China Hospital, Sichuan University, Chengdu, Sichuan P.R. China; 2 Department of Pathology, West China Hospital, Sichuan University, Chengdu, Sichuan P.R. China

**Keywords:** Liver injury, MDSCs, Liver regeneration, Liver fibrosis, Cytokines

## Abstract

**Background:**

The liver regeneration is a highly complicated process depending on the close cooperations between the hepatocytes and non-parenchymal cells involving various inflammatory cells. Here, we explored the role of myeloid-derived suppressor cells (MDSCs) in the processes of liver regeneration and liver fibrosis after liver injury.

**Methods:**

We established four liver injury models of mice including CCl_4_-induced liver injury model, bile duct ligation (BDL) model, concanavalin A (Con A)-induced hepatitis model, and lipopolysaccharide (LPS)-induced hepatitis model. The intrahepatic levels of MDSCs (CD11b^+^Gr-1^+^) after the liver injury were detected by flow cytometry. The effects of MDSCs on liver tissues were analyzed in the transwell co-culture system, in which the MDSCs cytokines including IL-10, VEGF, and TGF-β were measured by ELISA assay and followed by being blocked with specific antibodies.

**Results:**

The intrahepatic infiltrations of MDSCs with surface marker of CD11b^+^Gr-1^+^ remarkably increased after the establishment of four liver injury models. The blood served as the primary reservoir for hepatic recruitment of MDSCs during the liver injury, while the bone marrow appeared play a compensated role in increasing the number of MDSCs at the late stage of the inflammation. The recruited MDSCs in injured liver were mainly the M-MDSCs (CD11b^+^Ly6G^−^Ly6C^high^) featured by high expression levels of cytokines including IL-10, VEGF, and TGF-β. Co-culture of the liver tissues with MDSCs significantly promoted the proliferation of both hepatocytes and hepatic stellate cells (HSCs).

**Conclusions:**

The dramatically and quickly infiltrated CD11b^+^Gr-1^+^ MDSCs in injured liver not only exerted pro-proliferative effects on hepatocytes, but also accounted for the activation of profibrotic HSCs.

**Supplementary Information:**

The online version contains supplementary material available at 10.1186/s12876-024-03245-4.

## Introduction

Unique to other organs, the liver is characterized by its strong ability to regenerate in response to injury [1]. Liver regeneration to rebuild lost hepatic tissue is a fundamental parameter of liver, depending on a group of liver existing mature cells, especially hepatocytes [2]. However, in patients with chronic liver disease, diminished hepatocyte regeneration leads to the replacement of lost cellular mass with a fibrotic matrix. Long-term injury to liver cells and consistent activation of the fibrogenesis can result in liver fibrosis which is characterized by altered liver architecture and impaired hepatocyte proliferation as well as excessive accumulation of extracellular matrix (ECM) [3].

Hepatocytes make up the majority of the liver’s cells—nearly 80% of all liver cells—while non-parenchymal cells take the remaining 20% [4]. The non-parenchymal portion of liver contains large amounts of Kupffer cells, hepatic stellate cells (HSCs), liver sinusoidal endothelial cells (LSECs), lymphocytes and non-resident immune cells [5]. Thus, the liver has been regarded as an immunological organ [6]. During the process of liver regeneration, the hepatocytes and non-parenchymal cells, including HSCs, Kupffer cells and recruited inflammatory cells collaborate extensively. Recently, the myeloid-derived suppressor cells (MDSCs), a heterogeneous population of myeloid cells, have been brought to the forefront of liver diseases [7]. The MDSCs are generally defined by the co-expression of myeloid-cell lineage differentiation antigen CD11b and Gr-1 (Ly6G/Ly6C). Based on cell morphology and phenotype, the MDSCs are commonly classified into two subsets, the granulocytic (G-MDSCs, CD11b^+^Ly6G^+^Ly6C^low^) and monocytic MDSCs (M-MDSCs, CD11b^+^Ly6G^−^Ly6C^high^), in which the M-MDSCs are relatively more suppressive in comparison with G-MDSCs [8, 9]. MDSCs mainly play an immunosuppressive role in the process of inflammatory infiltration. The nitric oxide (NO) generated by MDSCs could impede the toxicity of T cells through suppressing the MHCII transcription and promoting the apoptosis of T cells [10]. Moreover, the M-MDSCs, instead of G-MDSCs, are capable of activating the regulatory T cells (Tregs), thereby inhibiting the activations of CD4^+^ and CD8^+^ T cells, and natural killer (NK) cells [11, 12].

Growing evidences demonstrate the MDSCs are involved in regulating the immunoreaction in hepatic inflammation [13, 14]. In the hepatic immune microenvironment, the MDSCs could be recruited and differentiated by various mechanisms, containing interactions with other cell types and stimulation by soluble mediators. For instance, the HSCs could induce the differentiation of MDSCs from myeloid cells through the direct cell-cell interaction mediated by CD44 [15]. The human mesenchymal stromal cells activate the proliferation of MDSCs by releasing hepatocyte growth factor (HGF) [16]. Besides, MDSCs can also be recruited in liver by the soluble interleukin-6 (IL-6), thereby preventing the hepatocytes damage mediated by CD8^+^ T cell [17]. In the chronic liver injury, the activated HSCs promote the production of interleukin-10 (IL-10) by MDSCs, which in turn inhibits the profibrotic capacity of activated HSCs as well as promotes the proliferation of hepatocytes [18, 19]. Meanwhile, the MDSCs also secrete transforming growth factor-β (TGF-β), which could accelerate the liver fibrosis through activating the profibrogenic function of HSCs [20]. However, the precise functions of MDSCs in liver regeneration and how MDSCs impact cellular regeneration are still remain elusive.

To investigate the role of MDSCs in hepatic inflammation and regeneration, we established the mouse model of liver injury induced by carbon tetrachloride (CCl_4_). The damaged hepatocytes further release abundant free radicals, thus triggering the various non-parenchymal immune cells to yield a great diversity of cytokines with pro-inflammatory or anti-inflammatory functions that regulate the liver regeneration and fibrogenesis [21]. In this study, we revealed the enrichment of CD11b^+^Gr-1^+^ MDSCs in injured liver with secreting various cytokines to promote the proliferations of both hepatocytes and HSCs, which suggested the complicated roles of MDSCs in liver regeneration and fibrogenesis.

## Materials and methods

### Animals

Female BALB/c mice (6–8 weeks old) were obtained from the Huafukang Biotechnology Co. Ltd (Beijing, China) and housed in specific-pathogen-free (SPF) environment. Before experiments, all of the animals acclimatized for one week. According to the guidelines and protocols approved by the Animal Care and Treatment Committee of Sichuan University (Chengdu, China), all mice used in study were received with humanely treatment.

### Establishment of liver injury models

The CCl_4_ induced liver injury model was established as previously described [21]. The CCl_4_ was mixed with the olive oil in 1:2 ratio. Then, the mice were intraperitoneally injected with this mixed solution (2.5 µl/kg body weight) in a single dose, while the control-treated mice were injected with same volume of olive oil. BDL model, Con A-induced hepatitis model, and LPS-induced hepatitis model were also established as the following instructions. The surgical ligation of bile duct was conducted according to the previous protocol [22]. The Con A-induced hepatitis model was established by intravenously injecting ConA (20 mg/kg) in a single dose [23]. The mice were intraperitoneally injected with LPS (100 µg per mouse) for establishing LPS-induced hepatitis model [24]. At the indicated time points, the mice were anesthetized by abdominal injection with 60 mg/kg of 3% sodium pentobarbital. After the mice were confirmed with the complete unconsciousness, the liver tissues and bone marrows of mice were harvested for the subsequently experiments. All the animal experiments in this study were conducted in accordance with ARRIVE guidelines.

### Flow cytometry analysis of blood, bone marrow, spleen and intrahepatic MDSCs

The blood and bone marrow from the mice of established liver injury models were collected at indicated time points, followed by lysing the red blood cells under the pre-cooled red blood cell lysis buffer. For acquiring single cell suspension of spleen, we pushed the spleen through the 70 μm cell strainer. The obtained splenic cells were then subjected to red cell lysis. In addition, the livers of mice were perfused with phosphate buffer solution (PBS) and cut into small pieces, followed by being digested under the 1 mg/ml collagenase IV. After being filtered through the 70 μm cell strainer, the liver cell suspensions were subjected to red blood cell lysis. Subsequently, the single cells suspensions of blood, bone marrow, spleen and liver were prepared and stained with the FITC-conjugated rat anti-mouse CD11b and PE-conjugated rat anti-mouse Gr-1 antibodies at 4℃ for 30 min. These cells were then washed with PBS twice and used for flow cytometry analysis. Moreover, the prepared liver cell suspensions were additionally stained with APC-conjugated rat anti-mouse IL-10, PE-Cy7-conjugated rat anti-mouse VEGF, AF700-conjugated rat anti-mouse TGF-β, PerCP-Cy5.5-conjugated rat anti-mouse HGF, APC-Cy7-conjugated rat anti-mouse Ly6C and AF594-conjugated rat anti-mouse Ly6G for analyzing the phenotype of intrahepatic MDSCs.

### Serum biochemical tests

The blood of mice was harvested at indicated time points after the CCL_4_ injection. The blood was placed at 4℃ overnight, followed by centrifuging to obtain serum. Then, the concentrations of alanine aminotransferase (ALT) and aspartate aminotransferase (AST) in the obtained serum were detected by using an automatic biochemical analyzer (Hitachi High-Technologies Crop., Minato-ku, Tokyo, Japan).

### H&E staining and immunohistochemistry

For observing the histological structure of injured liver, the liver tissues of mice were harvested, and performed as paraffin-embedded sections. After being dewaxed and hydrated, the liver sections were stained with hematoxylin and eosin (H&E) for histomorphological observation. Moreover, the expressions of Gr-1, TGF-β, and vascular endothelial growth factor (VEGF) in liver tissues were assessed by immunohistochemistry staining (IHC). Firstly, the paraffin-embedded liver sections were dewaxed and hydrated, followed by blocking the endogenous peroxide with 3% H_2_O_2_ for 10 min. The epitope retrieval of sections was performed through heating in an autoclave. Then, the normal goat serum was used for blocking the non-specific binding at 37℃ for 30 min. After being incubated with specific primary antibody, the sections were washed with PBS. The sections were incubated with corresponding HRP-conjugated secondary antibodies at 37℃ for 30 min. Finally, the diaminobenzidine peroxide solution (DAB) was used to render color and the nuclei were counterstained with hematoxylin.

### Co-culture experiments

To investigate the effect of MDSCs on liver regeneration, the bone marrow-derived MDSCs were separated through magnetic-activated cell sorting (MACS) with the mouse myeloid-derived suppressor cell isolation kit (Miltenyi Biotec). First, the femur and fibula were isolated from normal mice. Both ends of bones were carefully trimmed, and the interior contents of bone marrow was collected followed by subjecting to red blood cell lysis. Then, the obtained liver cells were labeled with anti-Gr-1 Biotin and anti-Biotin microbeads. The labeled cells were loaded on the MACS column, within the magnetic field of the MACS separator. Thus, the Gr-1 positive cells were retained within the column and collected into the tubes. These cells were stained with FITC-conjugated rat anti-mouse CD11b and PE-conjugated rat anti-mouse Gr-1 antibodies and analyzed by flow cytometer in order to identify the purity of sorted cells. Subsequently, the liver tissues of normal mouse were dissected and cut into small pieces. The small pieces of liver tissue were placed in the lower chamber of transwell system, and the untreated medium (control), MDSCs derived from bone marrow, bone marrow cells and 293 cells were respectively seeded into the upper chamber, with a number of 1 × 10^5^ cells. The growth of liver tissues was closely observed and recorded. In detailed, the morphology of the liver tissues in the lower chamber was observed under the microscope at set time points (day 0, day 1, day 2, day 4, day 6, day 8, day 10, day 12 and day 14). Therefore, the percentage of liver tissues with cell proliferation and diameter of the cell growth out from the tissues were counted and measured at set time points. Moreover, the concentrations of TGF-β, VEGF, IL-10, and HGF in the co-culture system were detected by ELISA assay at day 0 and day 3 after the tissue planted, respectively. The mouse TGF-β, VEGF, and IL-10 ELISA detection kits were purchased from Thermo Fisher Scientific, and the HGF ELISA detection kit was purchased from R&D Systems. Furthermore, the specific antibodies of TGF-β, VEGF, IL-10 and HGF were respectively administrated into the low chamber of the co-culture system for blocking the corresponding cytokines, while the IgG was added as a control treatment. After adding specific antibodies, the growth of liver tissues in the co-culture system was frequently observed and recorded.

### Immunofluorescence

The cells growing out from the liver tissues were fixed in 4% paraformaldehyde for 15 min at room temperature, followed by permeabilizing with 0.1% triton X-100. After being blocked with goat serum for 30 min, the cells were incubated overnight with the primary α-smooth muscle actin (α-SMA) (Cell Signaling Technology, CST) or cytokeratin 18 (CK18) (Abcam) antibody at 4℃. Prior to imaging under the fluorescence microscope, the FITC-conjugated goat anti-rabbit IgG was used as the secondary antibody, and the nuclei were labeled with DAPI.

### Cell cycle assay

To identify the effect of MDSCs on the proliferation of hepatocytes. The primary hepatocytes were isolated from BALB/c mice, according to the previous protocol [25]. In briefly, the mouse was anaesthetized and the fur were wiped with 75% ethanol before incising the abdomen. To wash out the blood cells from liver, the inferior vena cave was cannulated and perfused with perfusion buffer (HEPES buffer with EDTA). Then, the liver was digested with perfusing collagenase (1 mg/ml) for digesting the collagen in the extracellular matrix, thus accelerating the dispersion of hepatocytes. The liver was gently dissected, and the gall bladder was removed. The sack of liver was torn and the cells in liver were gently released in the medium, followed by being filtered through the 70 μm cell strainer. The cells were collected by spinning at 50 × *g* for 2 min. Subsequently, the liver cells were re-suspended in the Percoll solution (Santa Cruz biotechnologies) for further purifying the isolated hepatocytes. Finally, the isolated hepatocytes from liver mice were counted and immediately plated for subsequent experiments. The MDSCs were sorting from the bone marrow through MACS using the mouse myeloid-derived suppressor cell isolation kit (Miltenyi Biotec). The supernatant from MDSCs was used as a conditional medium to stimulate the primary isolated hepatocytes, and the same amount RPMI-1640 medium was served as a control treatment. After 48 h treatment, the hepatocytes were collected and fixed overnight in 70% ethanol at 4℃. Later, the hepatocytes were stained under the propidium iodide solution (50 µg/ml) containing RNase A (20 µg/ml) for 30 min and analyzed by the NovoCyte Flow cytometer (ACEA Biosciences).

### BrdU staining assay

The primary isolated hepatocytes were seeded at the 6-well plate with supplementary of different concentrations of cytokines including TGF-β, VEGF, HGF and IL-10. These hepatocytes were maintained culturement with the respective cytokines for set time points (day 1, day 3, day 7, day 11 and day 15), and the BrdU (50 µM, Abcam) was added for 12 h befored the fixation. Then, these cells were fixed and immunostained with the BrdU antibody (1:2000, Abcam) according to the manufacturer’s instruction. Subsequently, the PE-coupled Rabbit anti-mouse secondary antibody (1:150, Abcam) was used for staining the BrdU-positive cells and the DAPI was used for nuclei counterstain. The proliferation ablility of hepatocytes were observed and photographed under the fluorescence microscopye (Olympus). Finally, the percentage of BrdU positive cells was evaluated at set time points.

### Statistical analysis

All statistical analyses in this study were conducted using the GraphPad Prism version 9.0 (GraphPad Software Inc.). When two groups were compared, the two-tailed unpaired Student’s *t* test was performed. The data were represented as mean ± standard error of mean (SEM). The differences were regarded as statistically significant when the *p* < 0.05. The significant data in the figures were expressed as: *p < 0.05, **p < 0.01, ***p < 0.001 and ****p < 0.0001.

## Results

### The CD11b^+^Gr-1^+^ MDSCs were recruited into injured liver

**Fig. 1:**
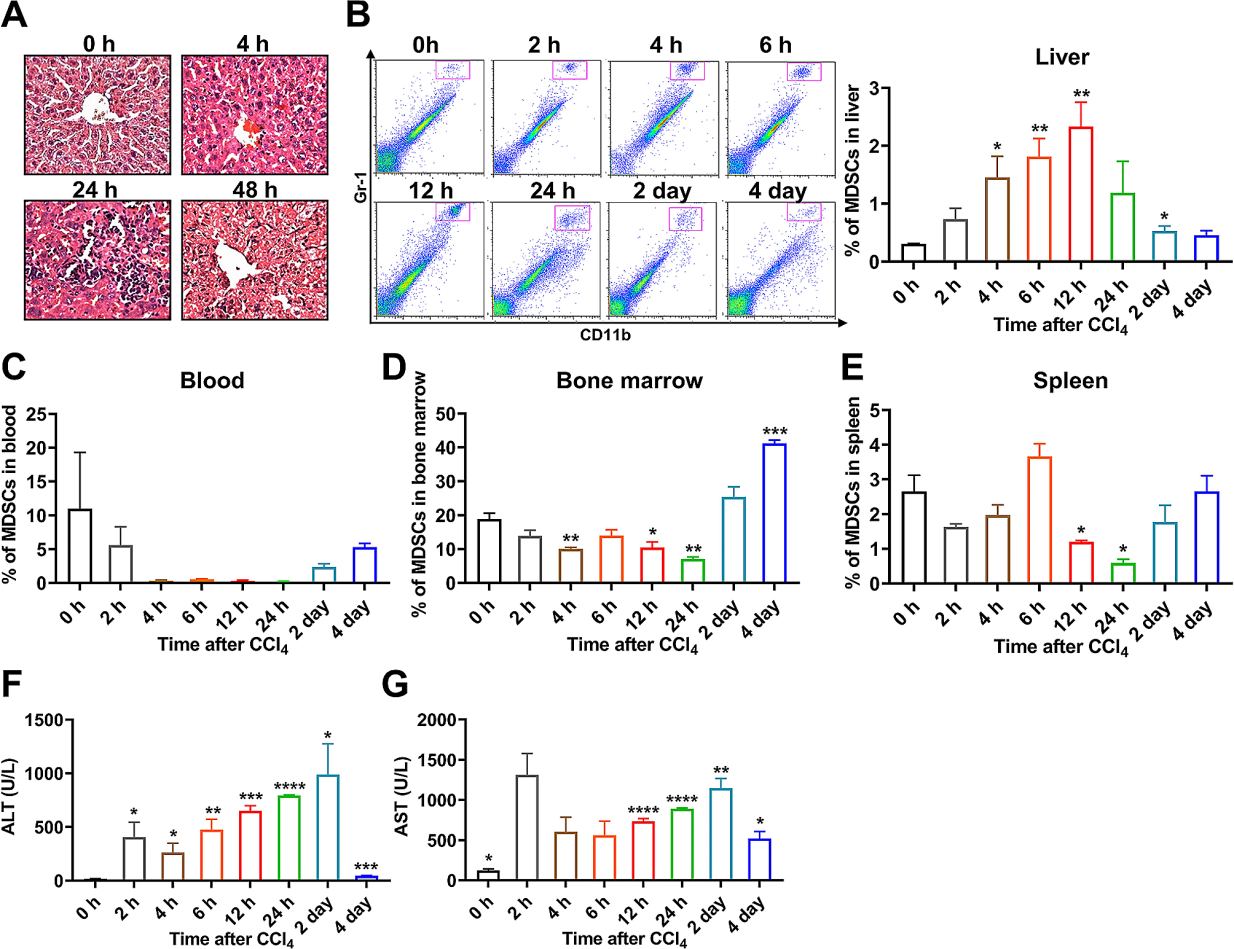
CD11b^+^Gr-1^+^ MDSCs were enriched within liver after the acute hepatitis injury. The mice were intraperitoneally injected with CCl_4_ solution. At the indicated times after injection, the liver, blood, bone marrow and spleen of mice were collected. **A** H&E staining of liver sections showed the increased infiltration of inflammatory cells into the liver after acute injury. **B** The proportions of intrahepatic MDSCs (CD11b^+^Gr-1^+^) after acute injury were detected using flow cytometry. **C-E** At the indicated times after CCl_4_ injection, the proportions of MDSCs in blood (**C**), bone marrow (**D**), and spleen (**E**) were evaluated by flow cytometry. **F-G** The serum levels of ALT (**F**) and AST (**G**) in mice at the indicated times after CCl_4_ injection. (*n* = 3). *p < 0.05, **p < 0.01, ***p < 0.001 and ****p < 0.0001

MDSCs have been largely investigated and demonstrated with immunosuppressive functions in variety of diseases in recent years [26]. However, roles of MDSCs in liver fibrosis upon hepatitis and the underlying mechanisms have not been well addressed. To investigated the characteristics of MDSCs infiltration during liver injury, We intraperitoneally injected CCl_4_ into BALB/c mice and induced the acute hepatic injuries in the mice. When compared with the liver of control-treated mice (0 h), the CCL_4_ injection caused prominent necrosis of hepatocytes and infiltration of inflammatory cells at 24 h and 48 h (Fig. 1A). As shown in Fig. 1B, the percentage of MDSCs on the total liver were measured through flow cytometry, which suggested increased recruitment of MDSCs with surface marker of CD11b^+^Gr-1^+^ in liver after acute injury. The proportion of recruited MDSCs cells in liver increased significantly from the onset of injection with a maximum at 12 h and then dropped to a stable proportion but slightly higher than the control level (Fig. 1B). The hepatic recruitment of MDSCs during hepatitis intrigued us to identify its roles in hepatitis. To identify compartmental reservoirs of MDSCs by screening candidate tissues for recruiting, we detected the proportions of MDSCs in the blood, bone marrow and spleen of control-treated mice and CCL_4_-treated mice by flow cytometry. Accompanied by the increase in liver, the proportion of MDSCs in blood decreased in the first place and the percentage remained decreased until 4 days after the CCl_4_ injection (Fig. 1C). There also showed a remarkably increase in the percentage of MDSCs in the bone marrow after 2 days, which came after a mild reduction in number from the onset of CCl_4_ injection (Fig. 1D). Meanwhile, the proportion of MDSCs in spleen displayed a moderate decline then an erratic fluctuation after the CCl_4_ injection (Fig. 1E). Collectively, these observations suggested that the blood was the main reservoir of hepatic recruitment of MDSCs during the liver injury, and bone marrow probably plays a compensated role in increasing the number of MDSCs at late phase of the inflammation. In consistent with the changes of MDSCs infiltration in liver, the serum levels of ALT and AST also rose gradually at the first 2 days but then declined after 4 days after CCl_4_ injection (Fig. 1F-G).

**Fig. 2:**
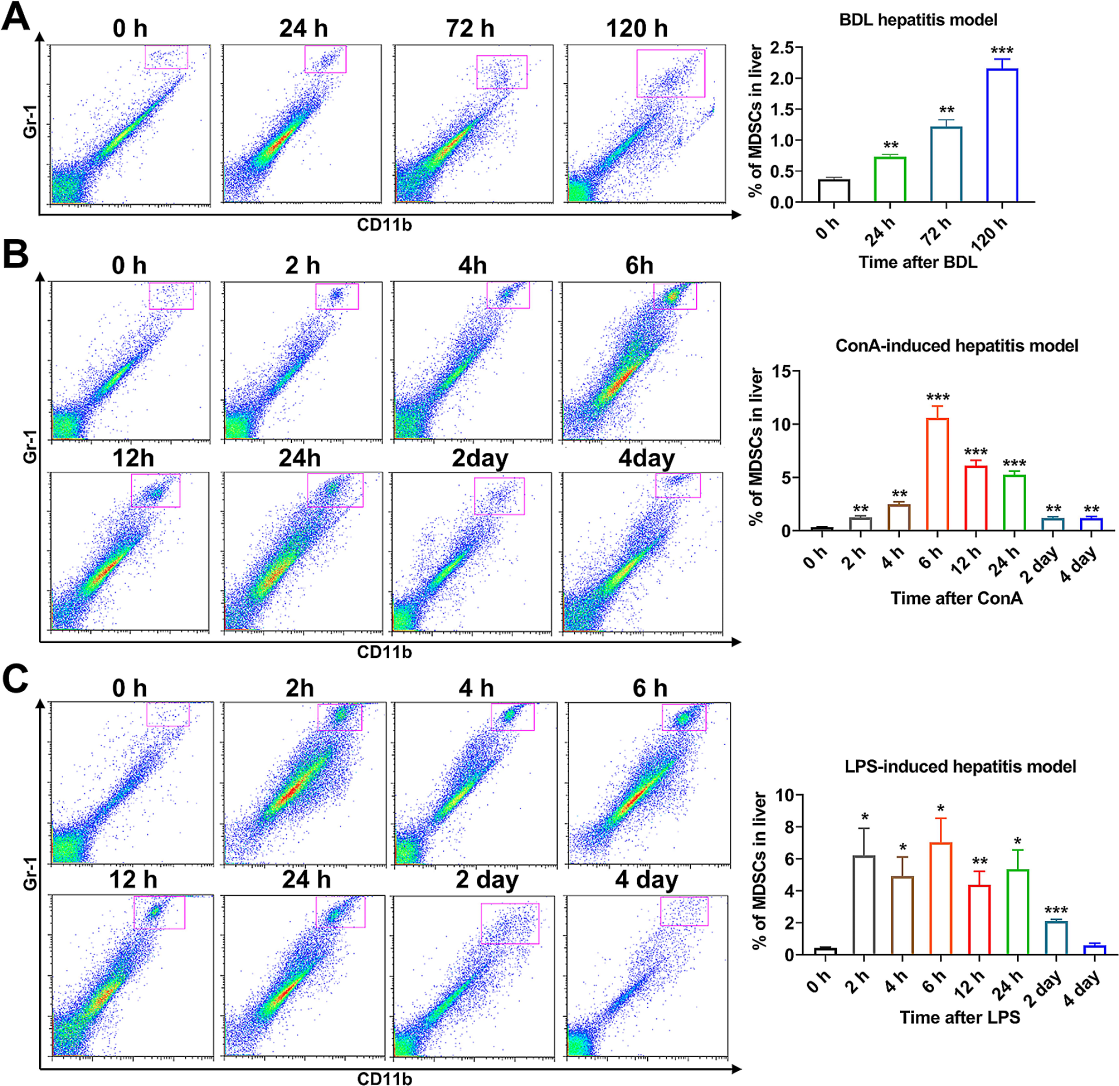
Increased intrahepatic MDSCs were discovered in other models of acute hepatitis. After receiving corresponding treatment, the various acute hepatitis models of mice were established. **A-C** The livers of mice from each hepatitis model were collected at set time points. The flow cytometry was used for analyzing the intrahepatic MDSCs (CD11b^+^Gr-1^+^) in mice of the BDL model (**A**), ConA-induced hepatitis model (**B**) and LPS-induced hepatitis model (**C**) (*n* = 3). *p < 0.05, **p < 0.01 and ***p < 0.001

Next, to verify whether the recruitment of MDSCs into injured liver was a general phenomenon or an exception, we examined the proportions of MDSCs after acute liver damage in other commonly used models of hepatitis including the BDL-induced, Con-A-induced and LPS-induced models. In the BDL-treated mice, the proportion of MDSCs kept rising during the first five days after the BDL (Fig. 2A). In Con-A induced hepatitis model, the amount of MDSCs peaked at 6 h and then resumed to a stable level that was slightly higher than usual at 2 days (Fig. 2B). In LPS-induced hepatitis model, the intrahepatic MDSCs also displayed a rapid increase at 2 h and continued to be abundent at 2 days after the injection of LPS (Fig. 2C). Taken together, different mouse liver injury model showed the same trend that the recruitment of MDSCs into the liver significantly increased when liver injury firstly occured.

### The recruited MDSCs in injured liver is featured with increased expressions of multiple cytokines

**Fig. 3:**
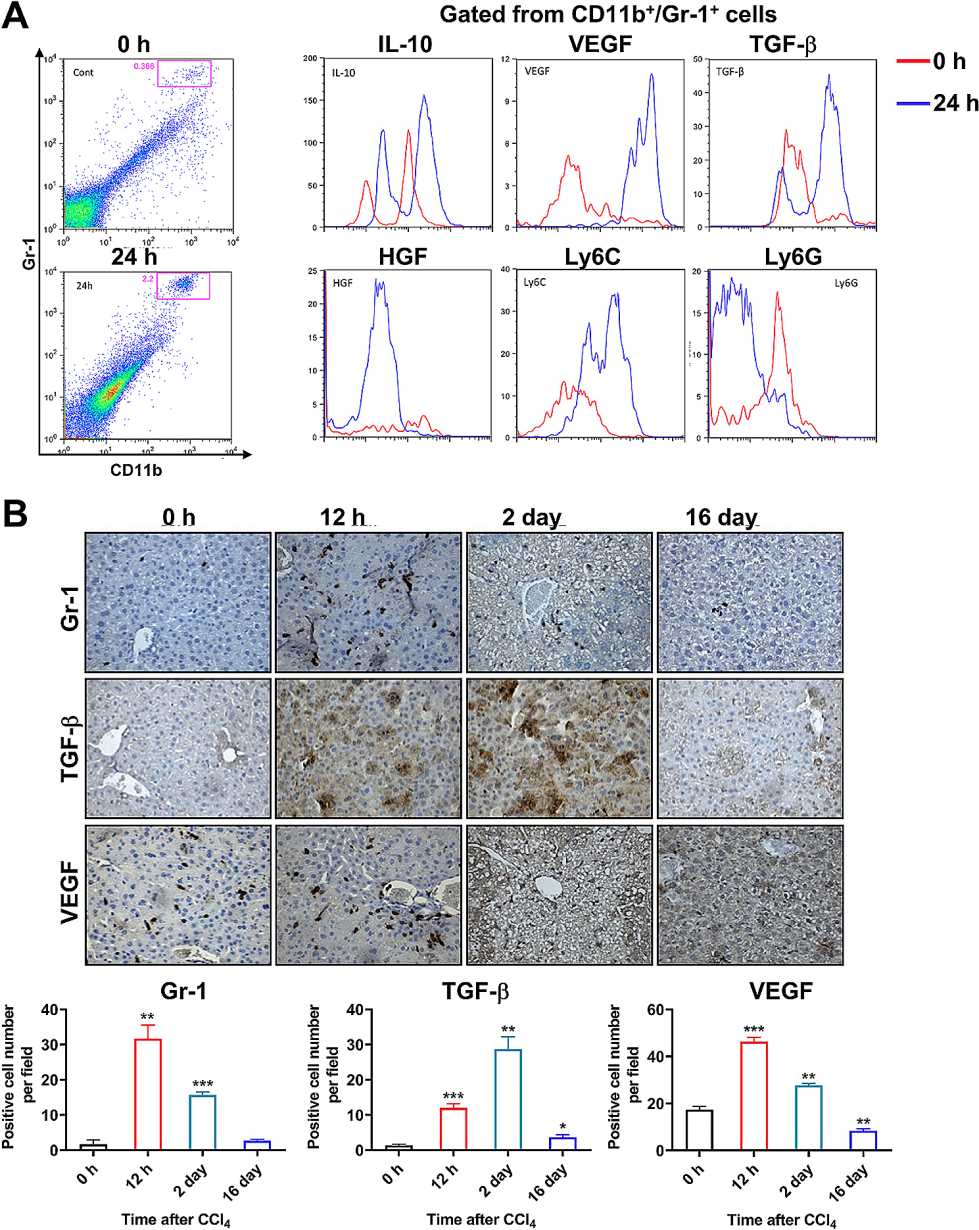
Characteristics of the hepatitis-related MDSCs. **A** The expressions of IL-10, VEGF, TGF-β, HGF, Ly6C, and Ly6G in MDSCs from livers of control-treated (0 h) or livers at 24 h after the CCl_4_ injection were evaluated by flow cytometry. **B** The expressions of Gr-1, TGF-β, and VEGF in the livers at indicated times after CCl_4_ injection were assessed by IHC staining. *p < 0.05, **p < 0.01 and ***p < 0.001

To further evaluate the functions of recruited MDSCs in the injured livers, we investigated the expressions of IL-10, VEGF, TGF-β and HGF in the MDSCs from normal and injured livers. When compared with the MDSCs in normal liver, the recruited MDSCs in injured liver showed relatively higher expressions of IL-10, VEGF, TGF-β and HGF (Fig. 3A). Higher levels of growth factors such as VEGF, and TGF-β and HGF have been proposed to promote the proliferation of hepatocytes and induce liver regeneration [30–34]. IL-10, as an immunosuppressant cytokine, could also suppress the inflammation during the liver injury and support the growth of hepatocytes. The MDSCs are commonly divided into two subsets, the granulocytic (G-MDSCs, CD11b^+^Ly6G^+^Ly6C^low^) and monocytic MDSCs (M-MDSCs, CD11b^+^Ly6G^−^Ly6C^high^), in which the M-MDSCs are relatively more suppressive in comparison with G-MDSCs. Therefore, we further examined the subtypes of the recruited hepatitis-related MDSCs by flow cytometry. As shown in Fig. 3A, the recruited hepatitis-related MDSCs mainly demonstrated a phenotype of high Ly6C and low Ly6G expressions in comparison with the non-hepatitis related MDSCs. Therefore, the recruited MDSCs in injured liver were mainly the mononuclear MDSCs (M-MDSCs, CD11b^+^Ly6G^−^Ly6C^high^). As displayed in Fig. 3B, IHC staining of liver tissues after injected with CCl_4_ indicated consistently dynamic tendencies among the infiltrations of MDSCs (Gr-1^+^), TGF-β and VEGF.

### The MDSCs promote the proliferation of hepatocytes and HSCs

**Fig. 4:**
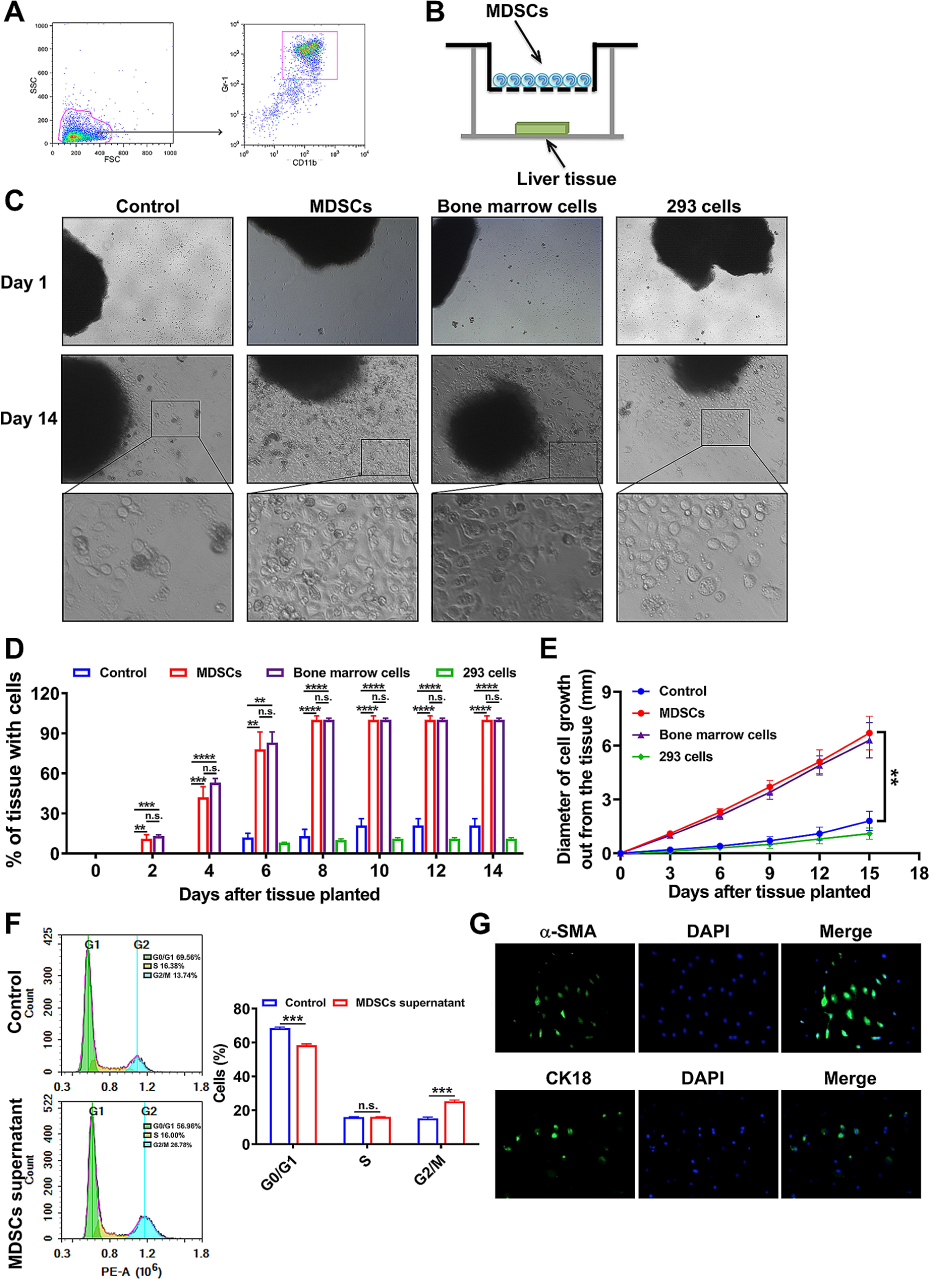
The MDSCs could promote the proliferation of hepatocytes. **A** The MDSCs (CD11b^+^Gr-1^+^) were isolated from the bone marrow of untreated mice by MACS. **B** The culture medium (control), MDSCs separated from bone marrow, bone marrow cells or 293 cells were respectively co-cultured with the normal liver tissues in vitro. **C** The morphology of the liver tissues in the co-culture system was observed under the microscope at day 1 and day 14. **D** The percentage of the liver tissues with cell growth after the co-culture at set time points. **E** The diameter of cell growth out from the liver tissues after the co-culture at set time points. **F** The cell cycles of primary isolated hepatocytes after being cultured with supernatants from MDSCs were detected through flow cytometry. **G** Representative immunofluorescence images of cells proliferated from the liver tissues that co-cultured with MDSCs at day 14. The α-SMA, CK18 and DAPI were stained. **p < 0.01 and ***p < 0.001; n.s., no statistical significance

Based on the observations that the recruited MDSCs at the liver injury displayed high expression levels of IL-10, VEGF, TGF-β and HGF, we proposed that the MDSCs promoted the growth of hepatocytes. To verify our hypothesis, the MDSCs from the bone marrow of normal mice were isolated by MACS. Then, the culture medium (control), MDSCs, primary isolated bone marrows cells of normal mice, and 293 cells were respectively seeded in the upper chamber and co-cultured with the normal liver tissues in a transwell system (Fig. 4A-B). The growth of the liver tissues in the lower chamber was examined and the percentage of the liver tissues with cell proliferation and diameter of the cell growth out from the tissues were counted and measured. As shown in the Fig. 4C, the co-culture with MDSCs or bone marrow cells significantly promoted the growth of cells, and some cells were equipped with typical morphological characteristics of hepatocytes out from the edge of liver tissue. Meanwhile, the co-culture with the MDSCs or bone marrow cells obviously promoted the probability and speed of cell growing out from the liver tissues (Fig. 4D-E). To further estimate the pro-proliferative effect of supernatants of MDSCs on hepatocytes, we isolated the primary hepatocytes from the liver of mouse, and the MDSCs were also sorted from the bone marrow through MACS. After the 48 h culturing of supernatants from MDSCs, the cell cycle of hepatocytes were detected. The supernatants of MDSCs significantly decreased the proportion of hepatocytes in G0/G1 phase and increased the proportion of hepatocytes in G2/M phase, which suggested the substances from MDSCs stimulated the hepatocytes proliferation (Fig. 4F). We have also imaged the cells grown out from the liver tissues that co-cultured with MDSCs by the HSCs marker α-SMA and the hepatocyte marker CK18 (Fig. 4G). Notably, we have observed comparable fluorescence signals of both α-SMA and CK18 in the proliferated cells, suggesting these cells growing out of the liver pieces contain not only hepatocytes but also HSCs. Thus, these results indicated that the MDSCs could not only promote the liver regeneration, but also facilitate the fibrogenesis during liver inflammation.

### MDSCs promoted the liver regeneration through secreting growth factors

**Fig. 5:**
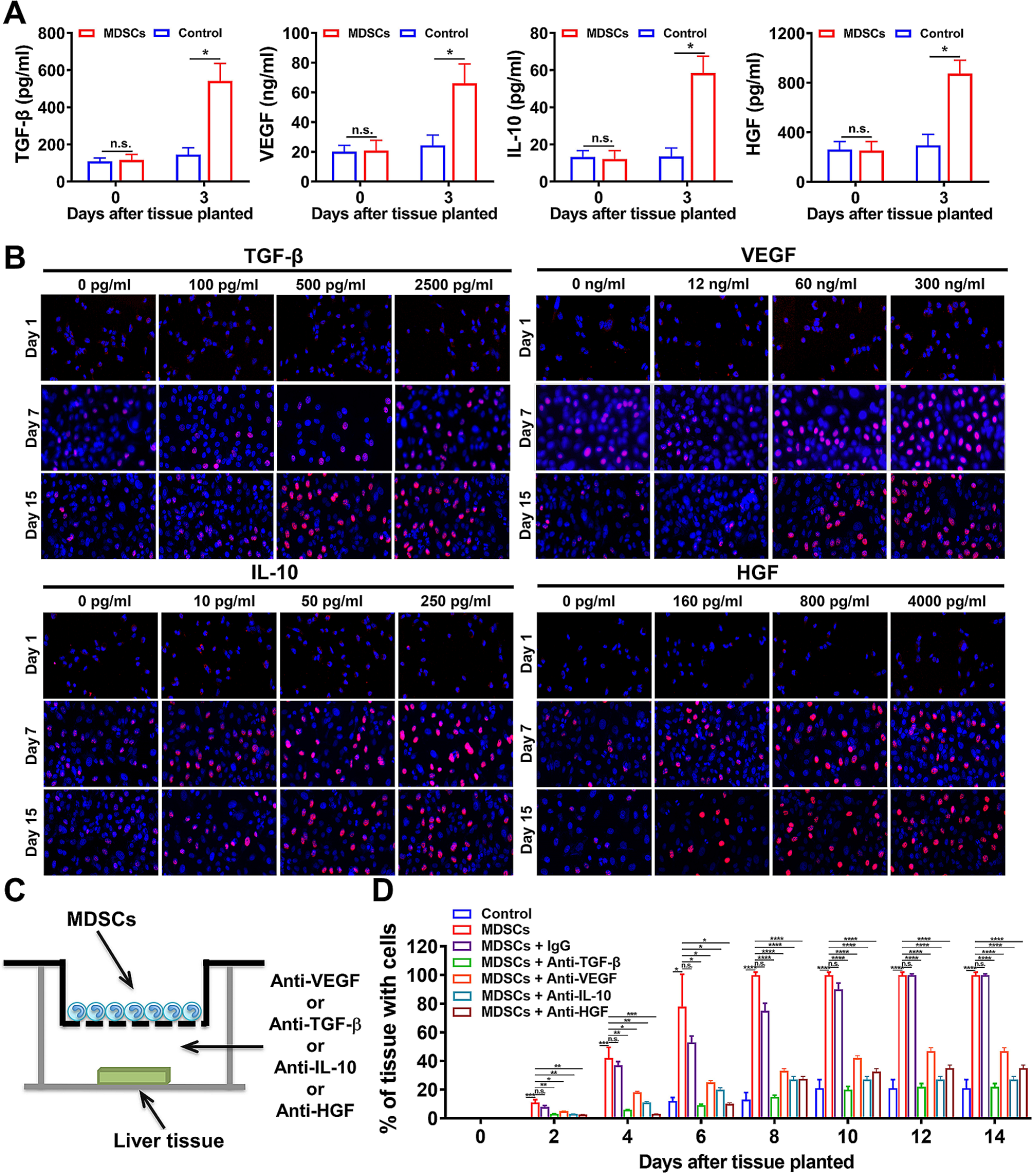
The cytokines secreted by MDSCs promoted the proliferation of hepatocytes. **A** The concentrations of TGF-β, VEGF, IL-10 and HGF in the supernatants of the co-culture medium were measured by ELISA assay. **B** The proliferation ability of primary isolated hepatocytes was promoted by the supplementary of TGF-β, VEGF, IL-10 and HGF, as measured by the BrdU staining assay. **C** The culture medium (control) and MDSCs were co-cultured with liver tissues, with or without specific antibodies. **D** The percentage of the liver tissues with cell growth after the co-culture at set time points. *p < 0.05, **p < 0.01, ***p < 0.001 and ****p < 0.0001; n.s., no statistical significance

To elucidated the cytokines secreted by recruited MDSCs in hepatitis, the supernatants of the co-culture system were collected and quantified by ELISA assay. Compared with the culture medium (control treatment), the MDSCs co-culture demonstrated with significantly higher levels of TGF-β, VEGF, IL-10, and HGF (Fig. 5A). Furtermore, the addition of these cytokines including TGF-β, VEGF, IL-10, and HGF all significantly promoted the proliferation ability of primary isolated hepatocytes, as measured by the BrdU staining assay at set time points (Fig. 5B and Supplementary Fig. 1). Then we added specific antibodies targeted to these cytokines into the lower chamber of the co-cultured transwell system (Fig. 5C). The growths of cells out from liver tissues were significantly blocked by administrating specific antibodies of TGF-β, VEGF, IL-10 and HGF (Fig. 5D). These results collectively demonstrated that MDSCs, after their recruitment into the injured liver, secreted high concentrations of TGF-β, VEGF, IL-10 and HGF, thereby promoting the proliferation of α-SMA positive HSCs and hepatocytes.

## Discussion

Upon liver injury, the balance between liver regeneration and fibrogenesis depends on the complicated crosstalk of various cell types, involving hepatocytes, HSCs and recruited inflammatory cells. The accumulated MDSCs in liver have attracted considerable attentions for their crucial role as an essential regulator in liver inflammation. In this investigation, we observed a prominent and quick accumulation of CD11b^+^Gr-1^+^ MDSCs after acute liver injury. The recruitment of intrahepatic CD11b^+^Gr-1^+^ MDSCs was mainly derived from the pool of peripheral blood, and the bone marrow served as a reserve in increasing the intrahepatic MDSCs at late phase of inflammation.

The increased infiltration of MDSCs into liver has been generally observed in multiple liver diseases, including viral hepatitis, autoimmune hepatitis, and hepatocellular carcinoma [27–29]. We have demonstrated the substantially increased accumulation of CD11b^+^Gr-1^+^ MDSCs in liver of four well-established mouse models, containing CCl_4_-induced liver injury, BDL model, Con-A induced hepatitis model, and LPS-induced hepatitis model, which were also supported by previous studies [27–29]. In mice, the MDSCs (CD11b^+^Gr-1^+^) are commonly classified into two subtypes, the G-MDSCs and M-MDSCs, according to the diverse expressions of Ly6G and Ly6C [30]. The M-MDSCs resemble monocytes in phenotype and morphology, while the G-MDSCs resemble the neutrophils [31]. The distinct subtypes of MDSCs contribute to immunosuppression through diverse mechanisms. The M-MDSCs primarily yield large amounts of NO, arginase-1, and immunosuppressive cytokines including IL-10 and TGF-β to suppress T cells activation, whereas the G-MDSCs predominantly generate abundant reactive oxygen species (ROS) and deactivate T cells via tight cell-to-cell contact [31–33]. Considering the relative instability and short half-life of ROS in comparison with that of NO, arginase‐1 and cytokines produced by M-MDSCs, the M-MDSCs exert more powerful suppressive activity than that of G-MDSCs [34]. Our result showed the majority of recruited MDSCs in injured liver were the subpopulation of M-MDSCs.

Several findings in this study support that the recruited MDSCs contribute to both the liver regeneration and fibrogenesis. Meanwhile, the co-culture with recruited MDSCs also motivated the activation of HSCs, manifested with the expression of α-SMA. Furthermore, elevated cytokines including TGF-β, VEGF, IL-10, and HGF were detected in the co-culture system in the presence of MDSCs. Blocking these cytokines with specific antibodies significantly abolished the pro-proliferative effects of MDSCs. The mitogenic growth and angiogenic signals are crucial for driving the liver regeneration [35]. Thus, the HGF and VEGF produced by recruited MDSCs in injured liver could coordinate together to promote the liver regeneration. In chronic liver inflammation, the activated HSCs that trans-differentiated to myofibroblasts are the key contributor for fibrosis through generating abundant collagen [36, 37]. Noteworthy, the TGF-β has been proved to induce collagen synthesis through activating the trans-differentiation of HSCs into myofibroblast-like phenotypes [38–40]. Previous study has shown the in vitro treatment of TGF-β to hepatocytes could induce their apoptosis [41]. The current understanding of the exact roles of TGF-β in liver repair and liver fibrosis is indeed complex and somewhat equivocal. For instance, it has been suggested that TGF-β can have a dual role. At lower concentrations, TGF-β could exert cytostatic and apoptotic effects on hepatocytes, whereas TGF-β could lead to the activation of HSCs at higher concentrations [42]. In line with this, Yang et al. reported that short-term exposure (12 h) to TGF-β inhibited the activation of HSCs, while longer exposure (48 h) promoted HSCs activation [43]. We interpret these findings as possible evidence of context-dependent effects of TGF-β, depending on factors such as concentration and duration of exposure. In our co-culture study, we propose that MDSCs promote the activation of HSCs via a cocktail of different cytokines, which include TGF-β and IL-10. Further study is needed to fully elucidate these intricate interactions and their implications for liver repair. Therefore, upon the acute hepatitis environment, our findings together indicated the dual role of recruited MDSCs in both liver regeneration and liver fibrogenesis.

## Conclusions

The liver regeneration is a highly complicated process depending on the close cooperation between the hepatocytes and non-parenchymal cells involving various inflammatory cells. Likewise, the liver fibrosis as the general end-stage of liver diseases relays on the sustaining inflammatory status in which the HSCs and Kupffer cells are highly activated by cytokines such as TGF-β [44]. In summary, our studies demonstrated the dramatically and quickly infiltrated CD11b^+^Gr-1^+^ MDSCs in injured liver exerted the pro-proliferative effects on hepatocytes, but also accounted for the activation of profibrotic HSCs. These findings might be useful for better understanding about the role of MDSCs in liver diseases. Further explorations are necessary for ultimately clinical translation in chronic liver disease and fibrosis.

### Electronic supplementary material

Below is the link to the electronic supplementary material.

Supplementary Material 1

## Data Availability

All data generated or analysed during this study are included in this published article.

## Abbreviations

ALB: Albumin
ALP: Alkaline phosphatase
ALT: Alanine aminotransferase
AST: Aspartate aminotransferase
BDL: Bile duct ligation
CCl_4_: Carbon tetrachloride
CK18: Cytokeratin 18
Con A: Concanavalin A
DAB: Diaminobenzidine peroxide solution
ECM: Extracellular matrix
G-MDSCs: Granulocytic MDSCs
H&E: Hematoxylin and eosin
HGF: Hepatocyte growth factor
HSCs: Hepatic stellate cells
IHC: Immunohistochemistry staining
IL-10: Interleukin-10
IL-6: Interleukin-6
LPS: Lipopolysaccharide
LSECs: Liver sinusoidal endothelial cells
MACS: Magnetic-activated cell sorting
M-CSF: Macrophage colony-stimulating factor
MDSCs: Myeloid-derived suppressor cells
M-MDSCs: Monocytic MDSCs
NK cells: Natural killer cells
NO: Nitric oxide
PBS: Phosphate buffer solution
PMA: Phorbol 12-myristate 13-acetate
ROS: Reactive oxygen species
SEM: Standard error of mean
SPF: Specific-pathogen-free
TBIL: Total bilirubin
TGF-β: Transforming growth factor-β
TP: Total protein
Tregs: Regulatory T cells
VEGF: Vascular endothelial growth factor
α-SMA: α-Smooth muscle actin
