# Supplementary material for: Hepatic recruitment of myeloid-derived suppressor cells upon liver injury promotes both liver regeneration and fibrosis
Source: BMC Gastroenterol. 2024 May 14;24:163. doi: 10.1186/s12876-024-03245-4 (PMC11092103; doi:10.1186/s12876-024-03245-4)
Supplement: Supplementary file 1 — Supplementary Material 1 [file 12876_2024_3245_MOESM1_ESM.docx]

**Hepatic recruitment of myeloid-derived suppressor cells upon liver injury promotes both liver regeneration and fibrosis**

Qiongwen Zhang^1†^, Ting Yu^2†^, Huaicheng Tan^1^, Huashan Shi^1^*.

**Author affiliations:**

^1^ Department of Head and Neck Oncology, Cancer Center, and State Key Laboratory of Biotherapy, West China Hospital, Sichuan University, Chengdu, Sichuan, P.R. China

^2^ Department of Pathology, West China Hospital, Sichuan University, Chengdu, Sichuan, P.R. China

* **Corresponding author:** Huashan Shi, No. 17, Block 3, Southern Renmin Road, Chengdu, Sichuan 610041, People’s Republic of China; Email address: [shihuashan@scu.edu.cn](mailto:shihuashan@scu.edu.cn); Tel: +8618980606519; Fax: +2886621955.

^†^Qiongwen Zhang and Ting Yu contributed equally to this work.


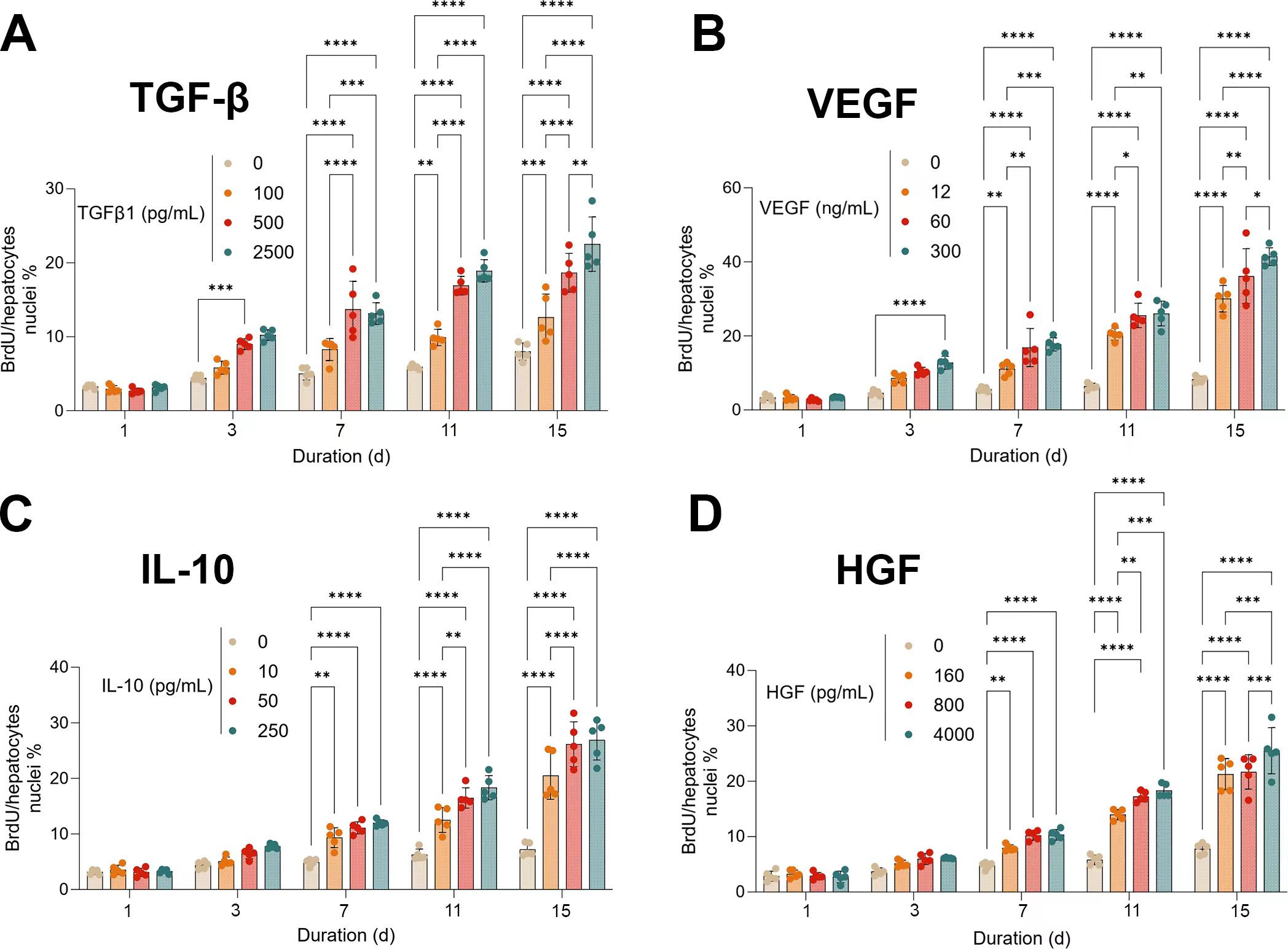


**Supplementary Figure 1.** The proliferation ability of primary isolated hepatocytes was promoted by the supplementary of TGF-β, VEGF, IL-10 and HGF, as measured by the BrdU staining assay. The proportions of BrdU positive hepatocytes were evaluated at set time points. **p* < 0.05, ***p* < 0.01, ****p* < 0.001, and *****p* < 0.0001.
